# Impact of albuminuria screening in primary care on the detection and management of chronic kidney disease: findings from the ONDAAS study

**DOI:** 10.1093/ckj/sfaf123

**Published:** 2025-04-25

**Authors:** Didier Sánchez-Ospina, Sebastián Mas-Fontao, Maria Martin Palencia, Badawi Hijazi Prieto, Cristina Gómez Sánchez, Silvia Paredes Carcedo, Maria Rodríguez Albo, Jesús Egido, Alberto Ortiz, Emilio Gonzalez-Parra, Maria Jesus Izquierdo Ortiz, ONDAAS study investigators, Vicente Villamandos Nicas, Nuria De la Fuente Esther Adrián, Teresa Mondejar Solís, Patricia Menéndez Rodríguez, Beatriz Campos Ruiz, Marta Sainz de Andueza, Luisa Natalia González Fernández, María Carmen Hernández Tuda, Marta Rodrigo Rodríguez, Azucena Bobadilla Alonso, Juan Ignacio Manzanal Bañuelos, Begoña Simón Serrano, Henar Conde Arce, Isabel Rábanos Oca, Fernando Fuertes García, Nuria Villamor Sagredo, Marina Gutiérrez Parra, Sara Gómez Burgos, Ángel Olea Movilla, Laura Alegre Ramos, Rut Sendino del Olmo, Ángel Minguito Pinedo, Manuel Parra Rivera, Amalia Sanz Sanz

**Affiliations:** Clinical Analysis Service, University Hospital of Burgos, Burgos, Spain; IIS-Fundación Jiménez Díaz, Autonoma University, Madrid, Spain; Diabetes and Associated Metabolic Diseases Networking Biomedical Research Centre (CIBERDEM), Madrid, Spain; Faculty of Medicine and Biomedicine, Universidad Alfonso X el Sabio (UAX), Madrid, Spain; Clinical Analysis Service, University Hospital of Burgos, Burgos, Spain; Division of Nephrology, Burgos University Hospital, Burgos, Spain; Ignacio Lopez Saiz Primary Care Center, Burgos, Spain; Primary Care Management of Burgos, Spain; Clinical Analysis Service, University Hospital of Burgos, Burgos, Spain; IIS-Fundación Jiménez Díaz, Autonoma University, Madrid, Spain; Diabetes and Associated Metabolic Diseases Networking Biomedical Research Centre (CIBERDEM), Madrid, Spain; Division of Nephrology and Hypertension, IIS-Fundación Jiménez Díaz, Universidad Autonoma de Madrid; IIS-Fundación Jiménez Díaz, Autonoma University, Madrid, Spain; Division of Nephrology and Hypertension, IIS-Fundación Jiménez Díaz, Universidad Autonoma de Madrid; IIS-Fundación Jiménez Díaz, Autonoma University, Madrid, Spain; Division of Nephrology and Hypertension, IIS-Fundación Jiménez Díaz, Universidad Autonoma de Madrid; Division of Nephrology, Burgos University Hospital, Burgos, Spain

**Keywords:** albuminuria, CKD, CKD-EPI equation, diabetic nephropathy, hypertension

## Abstract

**Background:**

The prevalence and burden of chronic kidney disease (CKD) is increasing. Despite available early detection methods, many individuals with CKD remain undiagnosed. We evaluate the effectiveness of albuminuria screening for early detection and management of CKD in the primary care setting.

**Methods:**

We conducted a cross-sectional, multicenter epidemiological study in primary care centers in the province of Burgos, Spain, from February to May 2024. The urinary albumin:creatinine ratio (uACR) was assessed in 9890 adults attending primary care visits. The primary finding was the prevalence of CKD defined by KDIGO guidelines thresholds for albuminuria and estimated glomerular filtration rate (eGFR), stratified by age, gender and motive for consultation.

**Results:**

In total, 22.29% of participants met CKD criteria, with 14.04% showing uACR levels above 30 mg/g (albuminuria categories A2–A3) and 12.81% displaying reduced eGFR levels (categories G3–G5). Albuminuria screening identified CKD in 1338 (14.1%) participants, including 903 (10.88%) individuals with eGFR >60 mL/min/1.73 m² and 434 (35.57%) participants with CKD upgraded to higher risk due to albuminuria. Among individuals with CKD, 2123 (22.29%) participants were eligible for therapies to slow disease progression, per current KDIGO guidelines.

**Conclusion:**

The ONDAAS study reveals a high prevalence of CKD among individuals attending primary care facilities, emphasizing the importance of albuminuria screening for early detection and risk stratification of CKD. Our findings demonstrate how early screening can significantly shape therapeutic choices and ultimately enhance patient care outcomes.

KEY LEARNING POINTS **What was known:**

- Chronic kidney disease (CKD) is prevalent but often undiagnosed in primary care settings, with many cases identified only in advanced stages.
- Albuminuria is a marker for both kidney and cardiovascular risks, yet it is frequently under-assessed in clinical practice, especially in early CKD stages.
- Prior studies highlight combining albuminuria with estimated glomerular filtration rate (eGFR) measurements could improve CKD detection and risk stratification.

**This study adds:**

- The ONDAAS study found a 22.4% prevalence of CKD in primary care, with albuminuria present in 14.1% of patients, suggesting substantial under-diagnosis when albuminuria screening is omitted.
- Albuminuria screening identified high-risk CKD cases that would not have been detected based on eGFR alone, influencing management decisions.
- This study underscores the need for routine albuminuria testing in primary care to detect early-stage CKD and improve patient outcomes.

**Potential impact**:

- Routine albuminuria screening could enable earlier interventions, reducing CKD progression and cardiovascular complications.
- Adoption of combined albuminuria and eGFR testing may influence guidelines for CKD management in primary care, aligning with KDIGO recommendations.
- Systematic screening in primary care could help identify high-risk patients, optimizing healthcare resources by targeting therapies to slow CKD progression.

## INTRODUCTION

Chronic kidney disease (CKD) is defined by persistent albuminuria or a low estimated glomerular filtration rate (eGFR, <60 mL/min/1.73 m²) for more than 3 months, as these parameters are associated with adverse health outcomes [1, 2]. Globally, over 850 million people suffer from CKD, and its prevalence is increasing [3]. Patients with CKD are more likely to progress to kidney failure which often necessitates dialysis or a kidney transplant. Even early-stage CKD is associated with a higher risk of cardiovascular disease and premature all-cause death [4]. Approximately 1 in 10 people worldwide is affected by CKD, and its prevalence increased by 29.3% from 1990 to 2017 [5]. Treating advanced CKD involves significant costs, consuming 2%–3% of annual healthcare budgets while covering less than 0.03% of population [6]. Additionally, life expectancy on kidney replacement therapy may be shorter by decades compared with the general population [7].

A systematic review reported an average CKD prevalence of 7.2% among individuals over 30 years of age in developed countries [8]. In Spain, the EPIRCE study found a CKD prevalence of 6.83% in stages 3–5, increasing to 21.42% for people over 64 years of age. When the urinary albumin:creatinine ratio (uACR) was added to diagnostic criteria, the general prevalence reaches 9.16% [9]. The CKD prevalence in Spain is comparable to other European countries (4.7%–8.1%) [10]. More recently, the ENRICA study (2018) found a CKD prevalence of 15.1% in adults from the general population, with stage 3a CKD being the most prevalent (10.0%) [11].

Early identification and treatment of CKD can slow its progression and prevent complications [12]. However, many individuals remain unaware of this condition [13, 14]. The KDIGO Controversies Conference advocated for systematic CKD detection, risk stratification and treatment, involving policymakers, local physicians and stakeholders in an iterative process [15]. CKD screening should include both eGFR and uACR assessments [16]. Nevertheless, many physicians do not test for albuminuria, even in patients with altered eGFR. Additionally, current guidelines recommend screening only those with established risk factors, potentially missing individuals without known risks [17].

Historically, CKD detection focused on eGFR, leading to later-stage intervention [18]. However, elevated albuminuria conveys a significant risk for cardiovascular events and CKD progression, even when eGFR is preserved [19]. Renin–angiotensin–aldosterone system inhibitors [angiotensin-converting enzyme inhibitors (ACEi)/angiotensin-II receptor blockers (ARBs)] are recommended for patients with moderate-to-severe albuminuria (A2–A3) to slow CKD progression and reduce cardiovascular risk. Separately, sodium-glucose cotransporter 2 (SGLT2) inhibitors have demonstrated benefits in CKD patients with and without diabetes, particularly in delaying disease progression and reducing mortality risk. Persistent pathological albuminuria is, by itself, a criterion to initiate therapy with these agents, according to both the Cardiology and Nephrology guidelines for cardiovascular disease prevention and CKD treatment [20–22]. Indeed, early treatment (i.e. when albuminuria is pathological but eGFR remains >60 mL/min/1.73 m^2^) may delay the need for kidney replacement therapy for up to 27 years [23]. Therefore, population-wide albuminuria screening has been suggested to identify early-stage CKD, enabling timely preventive treatments [24, 25]. Given the global CKD prevalence, primary care physicians play a critical role in early CKD detection, treatment and monitoring within an interdisciplinary framework also involving nephrologists and clinical biochemists.

This study explored population wide screening for adults attending primary care by adding uACR assessments to other biochemistry assessments ordered by the primary care physicians, with the aim of detecting early kidney damage and cardiovascular risk.

## MATERIALS AND METHODS

### Study design and setting

The ONDAAS (Objective No Dialysis: Asymptomatic Albuminuria Screening) study (NCT06447038) is a cross-sectional, multicenter epidemiological study conducted among users of both urban and rural primary care centers in the province of Burgos, Spain. The healthcare system in Burgos, Spain, is fully integrated into the Spanish National Health System, ensuring universal healthcare access. Single urine samples were collected to determine the uACR from all individuals aged ≥18 years whose family physicians requested a laboratory analysis during the study period from 1 February 2024 to 31 May 2024. The only exclusion criteria were being <18 years of age or a consultation motive of urinary tract infection (UTI). Each patient was included in the analysis a single time to avoid duplication. To minimize potential biases, standardized laboratory procedures were used for uACR and eGFR measurements, and data were collected directly from electronic health records to reduce information bias. Additionally, confounding factors such as age, gender and comorbidities were considered in the statistical analysis. The study size was determined based on the population served by the primary care centers in the province of Burgos during the study period. All eligible individuals aged ≥18 years who attended primary care consultations and met the inclusion criteria were included, ensuring a representative sample of the local population. No formal sample size calculation was performed, as the study aimed to capture real-world data from routine clinical practice.

### Sample collection and handling

During the primary care consultation, patients were provided with a sterile, hermetically sealed container for urine collection. Urine samples were handed to staff responsible for blood extraction, stored refrigerated and transported to the central laboratory at the University Hospital of Burgos, following the sample procedures manual. All procedures complied with the quality standards of UNE-EN ISO 9001:2015. The study protocol was approved by the Clinical Research Ethics Committee of the University Hospital of Burgos with number CEIm 3139, which waived informed consent.

### Renal function assessment

**Figure 1:**
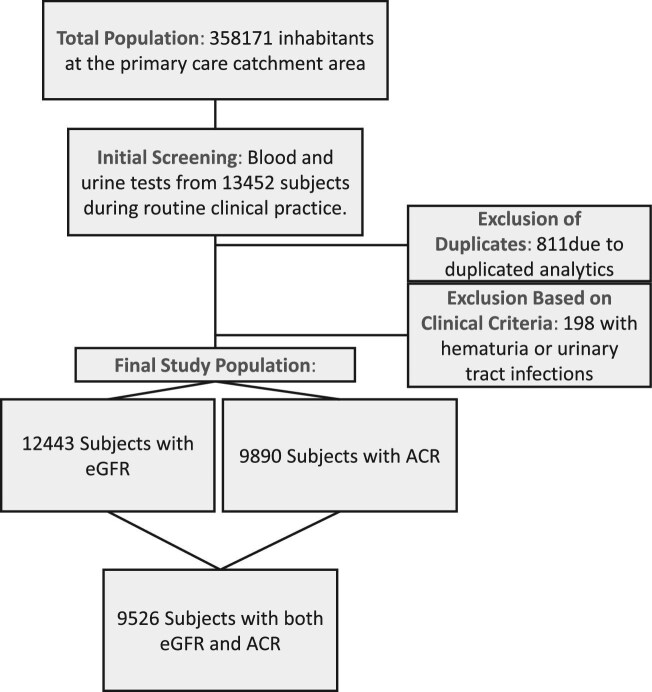
Flowchart illustrating the selection of subjects and the analysis of blood tests, showing exclusions and final counts for eGFR and uACR measurements.

Renal function was assessed according to the KDIGO 2024 Clinical Practice Guidelines, by testing uACR and eGFR. After excluding 811 individuals with duplicated analytics and 198 with suspected urinary tract infections or hematuria, a total of 12 443 subjects had eGFR measurements and 9890 had uACR measurements. The final analysis included 9526 participants with both eGFR and uACR measurements. Missing data were mainly attributed to individuals without eGFR or uACR measurements (Fig. 1).

CKD was classified into categories based on albuminuria and eGFR levels. Albuminuria categories include A1 (normal to mildly increased, <30 mg/g), A2 (moderately increased, 30–300 mg/g) and A3 (severely increased, >300 mg/g). GFR categories include G1 (normal renal function, ≥90 mL/min/1.73 m²), G2 (mildly decreased, 60–89 mL/min/1.73 m²), G3a (mild to moderate decrease, 45–59 mL/min/1.73 m²), G3b (moderate to severe decrease, 30–44 mL/min/1.73 m²), G4 (severe decrease, 15–29 mL/min/1.73 m²) and G5 (kidney failure, <15 mL/min/1.73 m²).

KDIGO categories were further characterized as mild, moderate or severe CKD, following the European Society of Cardiology and the European Renal Association simplified nomenclature for KDIGO risk categories [24].

### Laboratory analysis

Urinary albumin and creatinine levels were measured using a Cobas c 502 analyzer (Roche Diagnostics^®^). Urinary creatinine was assessed using the buffered kinetic Jaffé method without deproteinization, and urinary albumin was determined using an immunoturbidimetric method. The uACR was calculated using the laboratory's Infinity software (Roche Diagnostics^®^). The eGFR was calculated using the 2009 Chronic Kidney Disease Epidemiology Collaboration (CKD-EPI equations) [26] in a race-agnostic manner. Serum creatinine was determined using the Jaffé method with alkaline picrate, standardized on a Cobas c702 analyzer (Roche Diagnostics^®^).

### Patient monitoring and follow-up

The Infinity laboratory software (Roche Diagnostics^®^) enabled direct communication between laboratory physicians and primary care physicians through clinical diagnostic reports based on uACR results and eGFR. Identified patients with abnormalities in these parameters were followed up and intervened upon as necessary in subsequent studies.

### Variable definition

The variables considered in our study are the age and sex of the patients, as well as consultation motive, including hypertension, diabetes mellitus, prediabetes, metabolic syndrome, cardiovascular disease (CVD), dyslipidaemia, UTI, a previous diagnosis of CKD or other.

### Data collection

Demographic and clinical data were obtained from the Medora-CyL electronic health record system used in primary care facilities in Castilla y León and transferred to a coded Excel file. Analytical data were obtained from the Infinity laboratory information system (Roche Diagnostics) using the Omnium tool.

### Impact on therapy

The potential impact of the eGFR and uACR findings on therapy was estimated based on the KDIGO 2024 Clinical Practice Guideline for the Evaluation and Management of Chronic Kidney Disease (Supplementary Material) [20].

### Statistical analysis

Descriptive statistics were used to summarize patient demographics, clinical characteristics and laboratory results. Continuous variables were expressed as mean ± standard deviations (SD) or median and interquartile range (IQR) depending of the normality of the data, and categorical variables as frequencies and percentages. Comparisons between groups were made using Student's *t*-test for continuous variables and Chi-square test for categorical variables. Multivariable regression models were used to control for confounding factors such as age, gender and comorbidities. Missing data were handled by excluding cases with incomplete information, as the proportion of missing data was minimal (<5%). Subgroup analyses were performed to explore differences by age groups (<45, 45–64 and ≥65 years) and gender. Sensitivity analyses were conducted to assess the robustness of the results, including re-running the analyses after excluding participants with extreme values or potential outliers. All statistical analyses and graphs were performed using R using libraries ggplot2, tidyverse, dplyr and gamlss.

## RESULTS

### Study population characteristics

From a total population of 358 171 individuals in the Burgos healthcare area, 13 452 blood analyses were requested in routine clinical practice by primary care physicians during the study period from 1 February 2024 to 31 May 2024. In samples from 12 443 patients, serum creatinine was requested and the eGFR estimated and in urine samples from 9890 patients uACR was measured. A total of 9526 subjects had both eGFR and uACR measurements (Fig. 1). Of these, 198 were excluded because of suspected UTI or hematuria.

Demographic and clinical data for the study population are shown in Table 1. The age range spanned from 18 to 104 years with a median age of 61 years, and 5070 (41.8%) were men. Mean eGFR was 83.8 mL/min/1.73 m^2^ and median uACR 9 (5–18) mg/g. Dyslipidaemia (1702, 13.5%) diabetes (1483, 12.0%) and hypertension (1343, 10.6%) were common motives for consultation/follow-up both for men and women.

**Table 1:** Baseline characteristics of study population by gender.

Characteristics	Total (*N* = 12 134)	Female (*N* = 7064)	Male (*N* = 5070)	*P*-value
Edad				.017
Mean (SD)	60.9 (18.4)	60.6 (19.2)	61.4 (18.4)	
CKD-EPI				.002
Mean (SD)	83.8 (20.5)	84.2 (20.5)	83.1 (18.6)	
ACR				<.001
Median (IQR)	9 (5–18)	9 (5–18)	8 (4–18)	
Visit/follow-up reason				<.001
Diabetes	1483 (11.9%)	620 (8.5%)	863 (16.6%)	
Prediabetes	264 (2.1%)	135 (1.9%)	129 (2.5%)	
Dyslipidaemia	1702 (13.5%)	969 (13.2%)	733 (13.9%)	
Hypertension	1343 (10.6%)	692 (9.4%)	651 (12.3%)	
Metabolic syndrome	77 (0.6%)	25 (0.3%)	52 (1%)	
CVD	259 (2%)	121 (1.6%)	138 (2.6%)	
Preexisting CKD	164 (1.5%)	104 (1.4%)	82 (1.6%)	
UTI/hematuria	199 (1.6%)	115 (1.6%)	84 (1.6%)	
Other causes	7128 (57.3%)	4587 (63.2%)	2541 (49%)	

Student's *t*-test was used for comparing continuous variables (age, CKD-EPI and ACR) while Chi-square test was used for visit reason.

### Prevalence of CKD and distribution by CKD categories

CKD categories according to KDIGO used eGFR and albuminuria are shown in Table 2. Eighty-seven percent of participants had an eGFR ≥60 mL/min/1.73 m^2^. However, 265 (7.7%) of those in category G1 and 638 (13.2%) in category G2 had uACR values consistent with a diagnosis of CKD, usually in the 30–300 mg/g range.

**Table 2:** Distribution of CKD stages by KDIGO criteria using the CKD-EPI eGFR and albuminuria categories (in mg albumin per g creatinine).

		Albuminuria (ACR)			
CKD stage	eGFR CKD-EPI (mL/min/1.73 m^2^)	A1 (<30 mg/g)	A1 (30–300 mg/g)	A3 (>300 mg/g)	Total
G1	≥90	3199 (33.6%)	253 (2.7%)	12 (0.1%)	3464 (36.4%)
G2	60–89	4203 (44.1%)	603 (6.3%)	35 (0.4%)	4841 (50.8%)
G3a	45–59	561 (5.9%)	205 (2.2%)	28 (0.3%)	794 (8.3%)
G3b	30–44	188 (2%)	130 (1.4%)	22 (0.2%)	340 (3.6%)
G4	15–29	37 (0.4%)	41 (1.4%)	6 (0.1%)	84 (0.9%)
G4	<15		2 (0%)	1 (0%)	3 (0.1%)
Total		8188 (86%)	1234 (13%)	104 (1.1%)	9526 (100%)

Percentage values are expressed versus total patients with eGFR and ACR.

Among patients in eGFR category G3–G5 (<59 mL/min/1.73 m²), a majority was in CKD category G3a (8.3% of all participants), followed by category G3b (3.6%) (Table 2). The prevalence of A2–A3 albuminuria was higher in participants with eGFR G3–G5 than among those with eGFR G1–G2: 233 (29.3%) in G3a, 152 (44.5%) in G3b, 47 (56%) in G4 and 3 (100%) in G5. In this regard, assessment of ACR was informative at any eGFR category, either because it allowed to suspect a novel diagnosis of CKD (in G1–G2) or because it changed the risk category to a higher one, even for G3a and G3b, upgrading the risk to high or extremely high, a shift that had therapeutic consequences regarding guideline recommendations for treatment.

### Gender-specific impact of assessing albuminuria

CKD categories according to KDIGO used eGFR and albuminuria for men are shown in Supplementary data, Table S1 Eighty-eight percent of participants had eGFR ≥60 mL/min/1.73 m^2^. However, 127 (8.7%) of those in category G1 and 314 (14.4%) in category G2 had uACR values consistent with a diagnosis of CKD, usually in the 30–300 mg/g range.

Among patients in eGFR category G3–G5, a majority was in CKD category G3a (7.9% of all participants), followed by category G3b (3.3%) (Supplementary data, Table S1). The prevalence of A2–A3 albuminuria was higher in participants with eGFR G3–G5 than among those with eGFR G1–G2: 113 (34.5%) in G3a, 70 (51%) in G3b, 19 (70.4%) in G4 and 2 (100%) in G5. In this regard, assessment of ACR in men was also informative and had a therapeutic impact at any eGFR category.

CKD categories according to KDIGO used eGFR and albuminuria for women are shown in Supplementary data, Table S2 A total of 86.5% participants had an eGFR ≥60 mL/min/1.73 m^2^. However, 138 (6.9%) of those in category G1 and 324 (12.2%) in category G2 had uACR values consistent with a diagnosis of CKD, usually in the 30–300 mg/g range.

Among patients in eGFR category G3–G5, a majority was in CKD category G3a (8.7% of all participants), followed by category G3b (3,7%) (Supplementary data, Table S2). The prevalence of A2–A3 albuminuria was higher in participants with eGFR G3–G5 than among those with eGFR G1–G2: 120 (25.7%) in G3a, 81 (40.3%) in G3b, 28 (49%) in G4 and 1 (100%) in G5. In this regard, assessment of ACR in women was also informative and had a therapeutic impact at any eGFR category.

### Age-specific impact of assessing albuminuria

**Figure 2:**
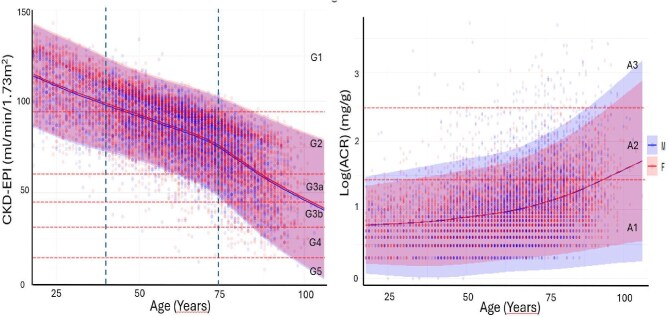
eGFR (left) and uACR (right) according to not age in a scatter plot.

Overall, eGFR decreased with increasing age (R value –0.707, *P* < .001) and an overlap was observed between men and women (Fig. 2A), while UACR values tended to increase with increasing age (Fig. 2B).

Table 3 depicts the distribution of CKD stages according to the CKD-EPI equation and the ACR across different age groups. CKD is primarily a condition affecting elderly individuals due to progressive decline in renal function. Among patients under 45 years (1428 individuals), 0.42% have an eGFR <60 mL/min/1.73 m², while 3.2% have A2–A3 albuminuria. However, 32 (2.8%) of those in category G1 and 11 (4.1%) in category G2 had uACR values consistent with a diagnosis of CKD, usually in the 30–300 mg/g range.

**Table 3:** Distribution of patients by KDIGO stages based on eGFR and ACR across age groups (18–44, 45–64, 65+ years).

		Age (years)			
eGFR KDIGO	mL/min/1.73 m²	18–44	45–64	65+	Total
G1	≥90	1831 (81.38%)	2227 (58.52%)	717 (12.68%)	4775 (39.58%)
G2	60–89	412 (18.31%)	1835 (44.10%)	3606 (63.78%)	5853 (48.51%)
G3a	45–59	6 (0.27%)	80 (1.92%)	844 (14.93%)	930 (7.71%)
G3b	30–44	1 (0.04%)	15 (0.36%)	384 (6.79%)	400 (3.30%)
G4	15–29	0 (0%)	4 (0.10%)	98 (1.73%)	102 (0.85%)
G5	<15	0 (0%)	0 (0%)	5 (0.09%)	5 (0.04%)
Total		2250 (100%)	4161 (100%)	5654 (100%)	12 058 (100%)
ACR KDIGO	mg/g	18–44	45–64	65+	Total
A1	<30	1381 (61.40%)	2989 (71.90%)	3814 (67.50%)	8184 (85.96%)
A2	30–300	40 (1.80%)	260 (6.25%)	933 (16.51%)	1233 (12.95%)
A3	>300	6 (0.27%)	15 (0.36%)	83 (1.47%)	104 (1.09%)
Total		1533 (100%)	3370 (100%)	4937 (100%)	9890 (100%)

Shows counts and percentages within each stage and age range for kidney disease severity analysis.

Among patients 45- to <65-year-old (3370 individuals), 2.63% have an eGFR <60 mL/min/1.73 m², while 8.4% have A2–A3 albuminuria. However, 151 (8.8%) of those in category G1 and 96 (6.5%) in category G2 had uACR values consistent with a diagnosis of CKD, usually in the 30–300 mg/g range (Supplementary data, Table S3).

Among patients or >65 years (4967 individuals), 23.4% have an eGFR <60 mL/min/1.73 m², while 21% have A2–A3 albuminuria. However, 82 (13.7%) of those in category G1 and 531 (17.1%) in category G2 had uACR values consistent with a diagnosis of CKD, usually in the 30–300 mg/g range (Supplementary data, Table S3).

As for the overall population and gender-based analysis, sensitivity analysis of the whole population having eGFR values or UACR values was consistent with results obtained in participants having both eGFR and UACR assessments.

### Linear regression analysis of eGFR with ACR and diagnosis

A linear regression model was performed to assess the relationship between eGFR, albuminuria (ACR), and diagnostic categories. The model (R² = 0.120) revealed that albuminuria (ACR) was significantly associated with lower eGFR (β = –0.0182, *P* < .001). Comorbidities such as dyslipidaemia (β = 4.5199, *P* < .001) and metabolic syndrome (β = 8.7030, *P* < .001) were associated with higher eGFR, suggesting potential hyperfiltration, while hypertension (β = –1.9483, *P* = .006), cardiovascular disease (β = –5.5073, *P* < .001) and CKD (β = –25.8290, *P* < .001) were linked to significantly reduced eGFR. Other conditions also showed a positive association with eGFR (β = 9.1042, *P* < .001). Despite these significant findings, the model explained only 12% of eGFR variability, indicating the influence of additional unmeasured factors.

### Potential impact on therapy

**Figure 3:**
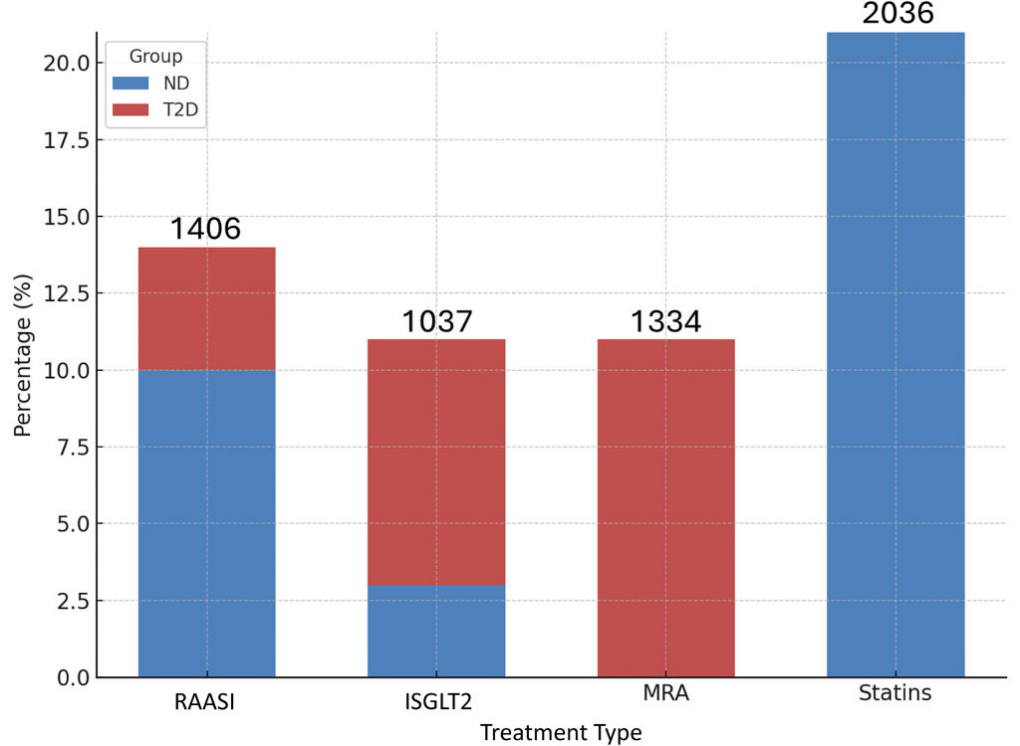
The potential impact on therapy was assessed based on KDIGO recommendations. Stacked bar chart showing the percentage usage of various treatments in patient groups with and without T2D.

The potential impact on therapy was assessed based on KDIGO recommendations [20]. Starting renin–angiotensin system (RAS) inhibitors (i.e. ACEi and/or ARBs) could be used in 1406 patients (14.6% of total). Of these, 69 were patients with CKD and severely increased albuminuria (G1–G4, A3) without diabetes (type 2 diabetes, T2D), 889 patients with CKD and moderately increased albuminuria (G1–G4, A2) without T2D, and 379 patients with T2D, CKD and moderately-to-severely increased albuminuria (G1–G4, A2 and A3) (Fig. 3).

Starting SGLT2 could be used in 1037 patients (10.89% of the total). Of these 782 patients with T2D, CKD and an eGFR ≥20 mL/min/1.73 m^2^, 104 were people without T2D and eGFR ≥20 mL/min/1.73 m^2^ with uACR ≥200 mg/g, and 151 were adults without T2D with eGFR 20–45 mL/min/1.73 m^2^ and uACR <200 mg/g (<20 mg/mmol) 3bA1-2.

Nonsteroidal mineralocorticoid receptor antagonist could be used in 1334 adults (14.01%) with T2D, an eGFR >25 mL/min/1.73 m^2^, normal serum potassium concentration and albuminuria (>30 mg/g).

Treatment with a statin or statin/ezetimibe combination could be used in 2036 patients (21.38%); of these, 1207 were adults aged ≥50 years with eGFR <60 mL/min/1.73 m^2^ (GFR categories G3a–G5), 818 adults aged ≥50 years with CKD and eGFR ≥60 mL/min/1.73 m^2^ (GFR categories G1–G2) and 11 adults aged 18–49 years with T2D, CKD (G3-4 and MA>30 mg/g) (Fig. 3).

In the therapeutic impact analysis, the following subgroups of patients were identified as eligible for specific treatments: 14.6% for RAS inhibitors, 10.89% for SGLT2 inhibitors, 14.01% for mineralocorticoid receptor antagonists and 21.38% for statins or statin/ezetimibe combinations.

## DISCUSSION

The primary findings of our study indicate that 22.29% of the individuals attending primary care physicians in the Burgos province, Northern Spain, have CKD according to KDIGO criteria, with albuminuria present in 14.1% of the samples. An annual eGFR decline of 0.85 mL/min/1.73 m² per year was also noted. Remarkably, 18.3% of individuals under 50 years of age had already lost some renal function, with eGFR between 60 and 90 mL/min/1.73 m². Although these individuals are not classified as having CKD, they require monitoring due to their increased risk of developing CKD in the future.

The average age in the population of Burgos province is 46.27 years, with 52.4% being women, compared with 61 years (with an age range from 18 to 104 years) and 58% in our study. This reflects a reasonably faithful representation of the study population. The age range allows for a comprehensive assessment of CKD prevalence across different life stages.

Previous population-wide screenings have shown varying CKD prevalence. For example, the EPIRCE study in Spain (2010) found a CKD prevalence of 6.8% in stages 3–5 using the Modification of Diet in Renal Disease 4 equation, with higher prevalence in older age groups [9]. Similarly, the ENRICA study (2018) reported a CKD prevalence of 11.9%, rising to 15.1% when including both eGFR <60 mL/min/1.73 m^2^ and elevated albuminuria [11]. The higher prevalence observed in our study (22.4%) may be attributed to the type of study (primary care vs general population screening), older average age (62 years) and higher presence of comorbidities. Population wide screening for albuminuria to detect early-stage CKD has not been extensively evaluated. For instance, the THOMAS study in the Netherlands investigated home-based screening methods for albuminuria detection, highlighting the need for more research on its utility and effectiveness [14]. Albuminuria is an independent predictor of cardiovascular risk, indicative of vascular injury and it is prevalent in 14.1% of our study population. One of the most important aspects of our study is that it uncovered a critical subgroup: 9.5% of individuals exhibited elevated ACR despite normal eGFR. This subset represents a population with high cardiovascular risk that otherwise would have gone unnoticed, and untreated [27]. That aligns with findings from other studies, such as the Canadian Health Measures Survey (2007–2009), which found a 10.3% prevalence of albuminuria among adults [28]. In this context, several studies have reported that in primary care setting the CKD prevalence rate is notably higher than in the general population, ranging from 15% to 27%, depending on the age and the conditions like diabetes and hypertension [29–31].

Our study highlights the essential role of primary care in CKD screening and management. In Spain, primary care serves as the first point of contact within the healthcare system, offering a critical opportunity for early detection. The ONDAAS study demonstrates that systematic albuminuria screening in primary care enables the identification of high-risk patients who might otherwise remain undiagnosed, especially those without traditional CKD risk factors. These findings underscore the necessity of structured CKD screening protocols in primary care to ensure timely nephroprotective interventions aligned with KDIGO guidelines. Additionally, early identification at the primary care level can alleviate the burden on specialized nephrology services, ensuring more efficient resource allocation and better long-term patient outcomes.

Our study also identified that 0.23% of the population had glomerular hyperfiltration [32], which plays a significant role in initiating glomerular damage, particularly in diabetic patients. Although hyperfiltration is primarily described in diabetes mellitus, it is also seen in conditions like hypertension, pregnancy and obesity/metabolic syndrome.

The study population's average age of 62 years showed a certain decline in eGFR of 0.85 mL/min/1.73 m² per year. This decline is consistent with the natural ageing process [33], which is associated with structural and functional kidney alterations [34], including tubular atrophy, glomerulosclerosis, interstitial fibrosis and arteriosclerosis. Studies have shown similar GFR declines in both the general population and in carefully selected healthy kidney donors [35]. In our study, we found that 18.3% of individuals under 50 years of age have reduced renal function (eGFR 60–90 mL/min), not yet classified as CKD. This group requires monitoring due to increased future CKD risk, despite not meeting current CKD criteria. These findings highlight the importance of early detection and intervention, even in younger populations traditionally considered at lower risk.

Contrary to some studies that observed a higher prevalence of albuminuria with ageing [36], interpreted as increased endothelial damage [37], our study did not find a strong relationship between age and albuminuria onset. The prevalence of albuminuria increases with age, but the correlation in our dataset was weak (R = 0.103). This suggests that factors other than age, such as comorbid conditions, may play a more significant role in albuminuria prevalence among older adults.

The definition of CKD, particularly the eGFR threshold of 60 mL/min/1.73 m², is debated [38]. Epidemiological studies have supported this threshold due to the increased risk of death or end-stage renal disease with eGFR below 60 mL/min/1.73 m². However, an universal threshold does not account for the physiological decline in GFR with ageing. An age-calibrated definition of CKD has been proposed [39], suggesting different eGFR thresholds based on age groups to better differentiate between age-related and disease-related eGFR variations. The rationale behind a higher GFR threshold in younger individuals is to account for their longer risk horizon, which increases their lifetime risk of developing CKD at a given GFR stage, while older adults with an eGFR <60 mL/min/1.73 m² are less likely to progress to end-stage kidney disease due to competing mortality risks.

The KDIGO guidelines conclude that a significant number of individuals in the study would benefit from pharmacological treatment to slow the progression of kidney disease and/or reduce cardiovascular risk. RAS inhibitors would be indicated for 14.6%, SGLT2 inhibitors for 10.89%, nonsteroidal mineralocorticoid receptor antagonists for 14.01% and statins for 21.38%. Data on SGLT2 inhibitors confirm a benefit on renal outcomes and cardiovascular mortality in patients with CKD. SGLT2 inhibitors on chronic eGFR slopes were consistent with benefit at any eGFR or uACR level, potentially delaying kidney replacement therapy by 2–27 years, depending on baseline eGFR [23].

A key and yet unresolved issue in nephrology is understanding how the definition of CKD varies with age, enabling the identification of individuals at risk of progression to end-stage kidney disease. This information is highly relevant both for clinicians and healthcare policymakers, as it facilitates improved healthcare planning. In recent years, evidence has shown that CKD screening is cost-effective in the adult population of the USA [40]. This further underscores the importance of implementing early detection strategies, tailored to individual risk factors and the demographic characteristics of the population.

Additionally, we propose that primary care settings, regardless of the reason for consultation, represent an ideal environment for the implementation of such screening. This approach allows for broad population-based screening, enabling the efficient use of healthcare resources to detect CKD at its earliest stages.

### Limitations

This study has several limitations that should be declared. Firstly, as our sample was drawn from a single region in Spain, the generalizability of our findings to other regions or countries may be limited. Secondly, our study population comprised individuals attending primary care consultations, which could potentially bias the sample towards those with chronic conditions or those more actively engaged in preventive healthcare. This limits its extrapolation to broader populations. Additionally, as a cross-sectional study, causality between albuminuria and CKD progression cannot be established. Finally, ONDAAS is an observational study, and we did not evaluate the long-term impact of albuminuria screening on clinical outcomes such as CKD progression, cardiovascular events or mortality. Future longitudinal and interventional studies are needed to assess the effectiveness of albuminuria screening strategies in improving patient outcomes.

## CONCLUSIONS

This study reveals a substantial prevalence of CKD (22.29%) among individuals attending primary care facilities in North Spain, underscoring the imperative for early detection and vigilant monitoring, particularly in high-risk groups such as older subjects and those with hypertension, diabetes, CVD and other clinical conditions. Overall, albuminuria testing identified 905 patients with CKD not identified by eGFR and added risk stratification information with consequences for therapeutic decision-making in 16.4% of patients with KDIGO recommendations. These results emphasize the key role of widespread albuminuria testing in primary care units and the importance of family physicians in combating the CKD burden.

## Supplementary Material

sfaf123_Supplemental_Files

## Data Availability

The data underlying this article will be shared on reasonable request to the corresponding author.
