# Supplementary material for: Impact of albuminuria screening in primary care on the detection and management of chronic kidney disease: findings from the ONDAAS study
Source: Clin Kidney J. 2025 Apr 25;18(5):sfaf123. doi: 10.1093/ckj/sfaf123 (PMC12102690; doi:10.1093/ckj/sfaf123)
Supplement: sfaf123_Supplemental_Files [file sfaf123_supplemental_files.zip › 1426 CKD-ONDAAS-Manuscript---Supp-data.docx]

**Supplementary data**

**KDIGO 2024 Clinical Practice Guideline for the Evaluation and Management of Chronic Kidney Disease**(20)

**RASi**

Recommendation 3.6.1: We recommend starting renin-angiotensin-system inhibitors (RASi) (angiotensin-converting enzyme inhibitor [ACEi] or angiotensin II receptor blocker [ARB]) for people with CKD and severely increased albuminuria (G1–G4, A3) without diabetes (1B).

Recommendation 3.6.2: We suggest starting RASi (ACEi or ARB) for people with CKD and moderately increased albuminuria (G1–G4, A2) without diabetes (2C).

Recommendation 3.6.3: We recommend starting RASi (ACEi or ARB) for people with CKD and moderately-to-severely increased albuminuria (G1–G4, A2 and A3) with diabetes (1B)

**iSGLT2**

Recommendation 3.7.1: We recommend treating patients with type 2 diabetes (T2D), CKD, and an eGFR ≥20 ml/min per 1.73 m2 with an iSGLT2 (1A).

Recommendation 3.7.2: We recommend treating adults with CKD with an iSGLT2 for the following (1A): eGFR ≥20 ml/min per 1.73 m2 with urine ACR ≥200 mg/g (≥20 mg/mmol)

Recommendation 3.7.3: We suggest treating adults with eGFR 20 to 45 ml/min per 1.73 m2 with urine ACR <200 mg/g (<20 mg/mmol) with an iSGLT2 (2B).

**nsMRA**

Recommendation 3.8.1: We suggest a nonsteroidal mineralocorticoid receptor antagonist with proven kidney or cardiovascular benefit for adults with T2D, an eGFR >25 ml/min per 1.73 m2, normal serum potassium concentration, and albuminuria (>30 mg/g [>3 mg/mmol]) despite maximum tolerated dose of RAS inhibitor (RASi) (2A).

**Statins**

Recommendation 3.15.1.1: In adults aged ≥50 years with eGFR <60 ml/min per 1.73 m^2^ but not treated with chronic dialysis or kidney transplantation (GFR categories G3a–G5), we recommend treatment with a statin or statin/ezetimibe combination *(1A)*.

Recommendation 3.15.1.2: In adults aged ≥50 years with CKD and eGFR ≥60 ml/min per 1.73 m^2^ (GFR categories G1–G2), we recommend treatment with a statin *(1B)*.

Recommendation 3.15.1.3: In adults aged 18–49 years with CKD but not treated with chronic dialysis or kidney transplantation, we suggest statin treatment in people with one or more of the following *(2A)*: diabetes mellitus,

**Supplementary data 2 (eAnnex).**

**ONDAAS (Objetive no dialysis: Asymptomatic albuminuria screening) Study Investigators: Vicente Villamandos Nicas; Nuria De la Fuente Esther Adrián; Teresa Mondejar Solís ; Patricia Menéndez Rodríguez ; Beatriz Campos Ruiz , Marta Sainz de Andueza ; Luisa Natalia González Fernández ; María Carmen Hernández Tuda ; Marta Rodrigo Rodríguez; Azucena Bobadilla Alonso; Juan Ignacio Manzanal Bañuelos; Begoña Simón Serrano; Henar Conde Arce; Isabel Rábanos Oca; Fernando Fuertes García; Nuria Villamor Sagredo; Marina Gutiérrez Parra; Sara Gómez Burgos; Ángel Olea Movilla; Laura Alegre Ramos; Rut Sendino del Olmo; Ángel Minguito Pinedo; Manuel Parra Rivera; Amalia Sanz Sanz.
