# Supplementary material for: Impact of albuminuria screening in primary care on the detection and management of chronic kidney disease: findings from the ONDAAS study
Source: Clin Kidney J. 2025 Apr 25;18(5):sfaf123. doi: 10.1093/ckj/sfaf123 (PMC12102690; doi:10.1093/ckj/sfaf123)
Supplement: sfaf123_Supplemental_Files [file sfaf123_supplemental_files.zip › 1426 supplementary tables.docx]

**Table S1**. Distribution of Chronic Kidney Disease (CKD) Stages by KDIGO criteria using the CKD-EPI estimated Glomerular Filtration Rate (eGFR) and Albuminuria Categories (in mg Albumin per g creatinine) in male population


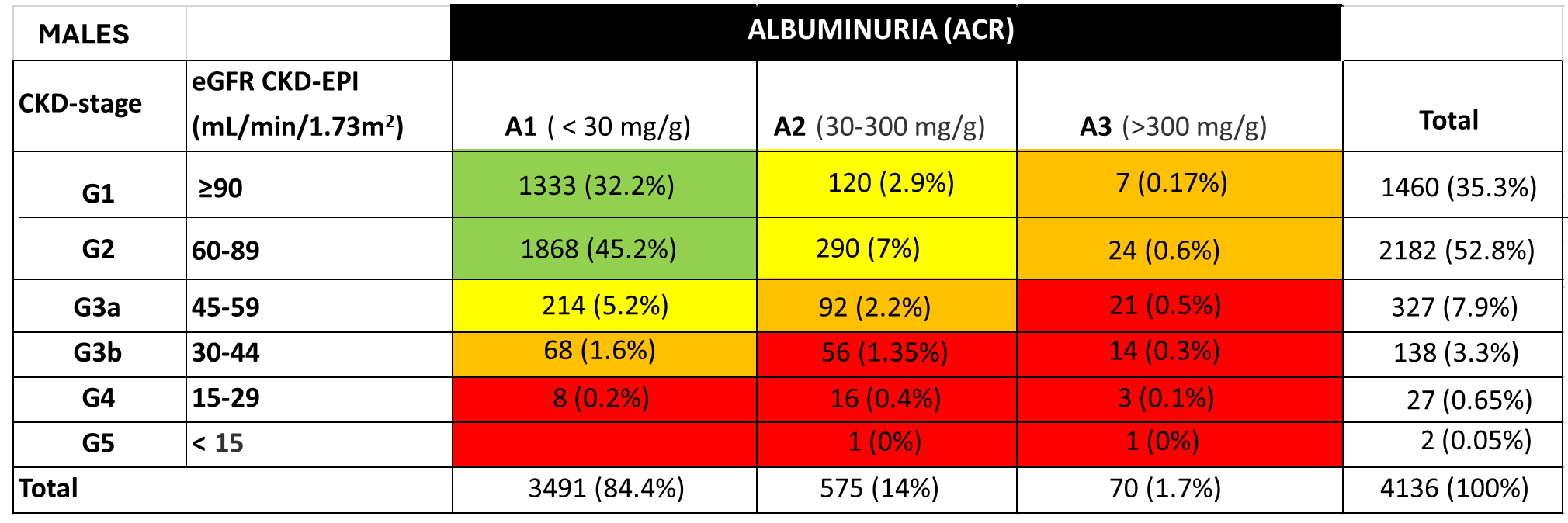


**Tables S2**. Distribution of Chronic Kidney Disease (CKD) Stages by KDIGO criteria using the CKD-EPI estimated Glomerular Filtration Rate (eGFR) and Albuminuria Categories (in mg Albumin per g creatinine) in female population


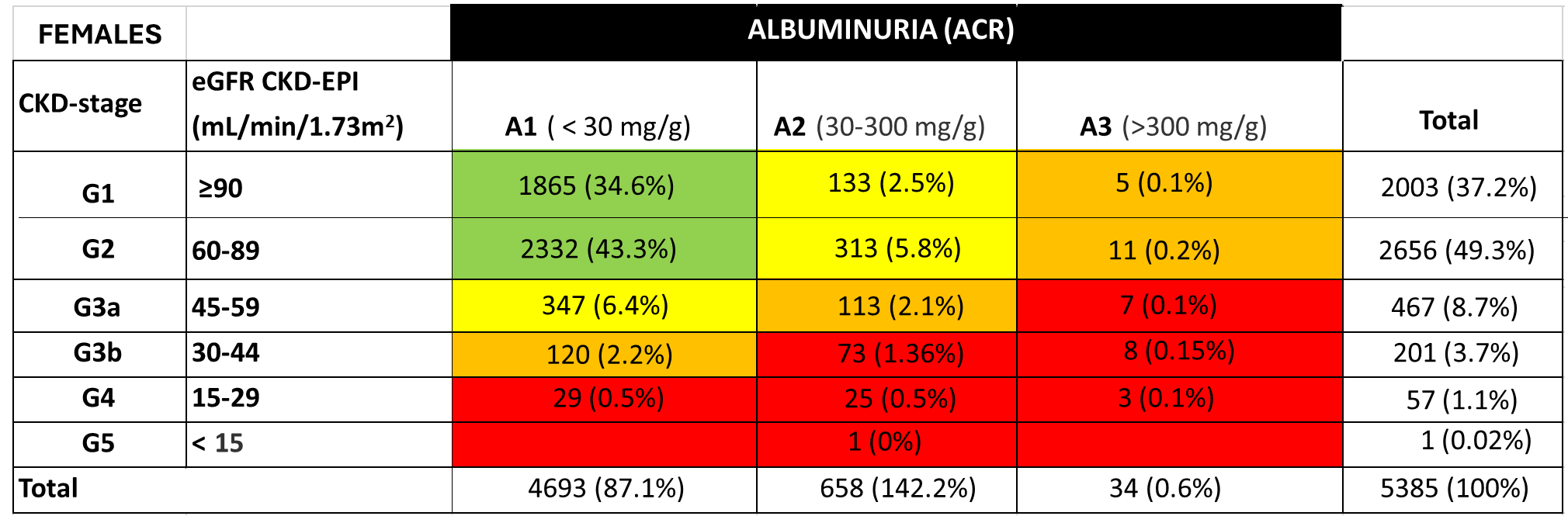


**Table S3**. Distribution of patients across age ranges (18-44, 45-64, and ≥65 years) and stages of chronic kidney disease (G1 to G5) categorized by albuminuria levels (A1, A2, A3).

| **Age Range: 18-44 Years** | | | | | |
| --- | --- | --- | --- | --- | --- |
| **eGFR KDIGO** | **(mL/min/1.73 m²)** | **A1 (ACR <30 mg/g)** | **A2 (ACR 30-300 mg/g)** | **A3 (ACR >300 mg/g)** | **Total** |
| **G1** | ≥90 | 1119 (78.36%) | 30 (2.10%) | 2 (0.14%) | 1151 (80.60%) |
| **G2** | 60-89 | 260 (18.22%) | 9 (0.63%) | 2 (0.14%) | 271 (19%) |
| **G3a** | 45-59 | 3 (0.21%) | 0 (0.00%) | 2 (0.14%) | 5 (0.35%) |
| **G3b** | 30-44 | 0 (0.00%) | 1 (0.07%) | 0 (0.00%) | 1 (0.07%) |
| **Total** |  | 1382 (96.78%) | 40 (2.80%) | 6 (0.42%) | 1428 (100%) |
| **Age Range: 45-64 Years** | | | | | |
| **eGFR KDIGO** | **(mL/min/1.73 m²)** | **A1 (ACR <30 mg/g)** | **A2 (ACR 30-300 mg/g)** | **A3 (ACR >300 mg/g)** | **Total** |
| **G1** | ≥90 | 1563 (47.87%) | 144 (4.41%) | 7 (0.21%) | 1714 (52.5%) |
| **G2** | 60-89 | 1369 (41.93%) | 93 (2.85%) | 3 (0.09%) | 1465 (44.87%) |
| **G3a** | 45-59 | 52 (1.59%) | 11 (0.34%) | 4 (0.12%) | 67 (2.05%) |
| **G3b** | 30-44 | 5 (0.15%) | 10 (0.31%) | 0 (0.00%) | 15 (0.46%) |
| **G4** | 15-29 | 1 (0.03%) | 2 (0.06%) | 1 (0.03%) | 4 (0.12%) |
| **Total** |  | 3074 | 279 | 17 | 3370 |
| **Age Range: 65+ Years** | | | | | |
| **eGFR KDIGO** | **(mL/min/1.73 m²)** | **A1 (ACR <30 mg/g)** | **A2 (ACR 30-300 mg/g)** | **A3 (ACR >300 mg/g)** | **Total** |
| **G1** | ≥90 | 517 (13.5%) | 79 (8.5%) | 3 (3.6%) | 599 (12.4%) |
| **G2** | 60-89 | 2574 (67.5%) | 501 (53.6%) | 30 (36.1%) | 3105 (64.2%) |
| **G3a** | 45-59 | 506 (13.3%) | 194 (20.8%) | 22 (26.5%) | 722 (14.9%) |
| **G3b** | 30-44 | 183 (4.8%) | 119 (12.7%) | 22 (26.5%) | 324 (6.7%) |
| **G4** | 15-29 | 36 (0.9%) | 39 (4.2%) | 5 (6%) | 80 (1.7%) |
| **G5** | <15 | 0 (0.00%) | 2 (0.2%) | 1 (1.2%) | 3 (0.1%) |
| **Total** |  | 3927 | 970 | 90 | 4967 |
